# SIRT7 Deacetylates STRAP to Regulate p53 Activity and Stability

**DOI:** 10.3390/ijms21114122

**Published:** 2020-06-09

**Authors:** Miao Yu, Xiaoyan Shi, Mengmeng Ren, Lu Liu, Hao Qi, Chi Zhang, Junhua Zou, Xiaoyan Qiu, Wei-Guo Zhu, Ying E. Zhang, Wengong Wang, Jianyuan Luo

**Affiliations:** 1 Department of Medical Genetics, Center for Medical Genetics, Peking University Health Science Center, Beijing 100191, China; aideen_yu@163.com (M.Y.); mengmengren23@163.com (M.R.); liulu_pku10@yufang.com (L.L.); phdqh@nus.edu.sg (H.Q.); zoujunhua2008@163.com (J.Z.); 2 Department of Biochemistry and Molecular Biology, Peking University Health Science Center, Beijing 100191, China; wwg@bjmu.edu.cn; 3 College of Pharmacy, Henan University, Kaifeng 475000, Henan, China; shisheep@126.com; 4 Department of Immunology, Key of Medical Immunology, Ministry of Health, School of Basic Medical Sciences, Peking University, Beijing 100191, China; azuretimm@bjmu.edu.cn (C.Z.); qiuxy@bjmu.edu.cn (X.Q.); 5 Department of Biochemistry and Molecular Biology, Shenzhen University School of Medicine, Shenzhen 518060, China; zhuweiguo@bjmu.edu.cn; 6 Laboratory of Cellular and Molecular Biology, Center for Cancer Research, National Cancer Institute, National Institutes of Health, Bethesda, MD 20892, USA; zhangyin@mail.nih.gov

**Keywords:** SIRT7, STRAP, p53, acetylation, 5-FU treatment

## Abstract

Serine-threonine kinase receptor-associated protein (STRAP) functions as a regulator of both TGF-β and p53 signaling that participates in the regulation of cell proliferation and cell death in response to various stresses. Here, we demonstrate that STRAP acetylation plays an important role in p53-mediated cell cycle arrest and apoptosis. STRAP is acetylated at lysines 147, 148, and 156 by the acetyltransferases CREB-binding protein (CBP) and that the acetylation is reversed by the deacetylase sirtuin7 (SIRT7). Hypo- or hyperacetylation mutations of STRAP at lysines 147, 148, and 156 (3KR or 3KQ) influence its activation and stabilization of p53. Moreover, following 5-fluorouracil (5-FU) treatment, STRAP is mobilized from the cytoplasm to the nucleus and promotes STRAP acetylation. Our finding on the regulation of STRAP links p53 with SIRT7 influencing p53 activity and stability.

## 1. Introduction

Serine/threonine kinase receptor associated protein (STRAP), known as the transforming growth factor-β1 (TGF-β) signaling negative regulator protein, is a 38kD protein that contains a WD40 domain [1]. The WD40 motif exists in a large number of proteins with regulative function, is involved in the interaction between proteins, and contains important effects in multiple biological processes, such as signal transduction, protein transportation, chromosome modification, transcription, and RNA processing [2,3]. It has been reported that STRAP binds to SMAD family member 7 (Smad7) and synergistically inhibits downstream signaling of TGF-β receptors, as a negative regulator of the TGF-β pathway [4]. In addition, recent studies have reported that STRAP is involved in the regulation of multiple signal transduction pathways, such as the canonical WNT/β-catenin, Apoptotic signal-regulating kinase 1 (ASK1), phosphatidylinositol-3-kinase (PI3K)/PDK, and notch receptor 1 (NOTCH) pathways, thereby regulating cell proliferation and apoptosis [5,6,7,8,9]. Furthermore, STRAP knockout mice showed high embryonic lethality during embryonic development because of defects in angiogenesis, neural tube closure, cardiac development, and embryonic turning, indicating that STRAP is required for normal embryo development [10]. Besides, TGF-β signaling has participated in p53-mediated signaling, in which p53 interacts with Smads proteins to regulate TGF-β gene responses [11,12]. STRAP has also been found to directly interact with p53 to enhance p53-induced apoptosis [13]. These reports indicate that STRAP might play a dual role in cellular processes. The mechanism of STRAP regulation remains elusive yet.

Sirtuin7 (SIRT7) is a member of the sirtuins, which has Nicotinamide adenine dinucleotide (NAD+)-dependent histone deacetylase activity [14]. More and more studies have indicated that SIRT7 participates in regulating ribosome biogenesis, cell proliferation, maintaining genome integrity, and metabolic homeostasis [14,15,16,17]. SIRT7 selectively removes acetyl from histone H3 lysine 18 (H3K18) as a lysine deacetylase [14]. Additionally, more SIRT7 deacetylase substrates have been reported, such as RNA polymerase I subunit E (PAF53), GA binding protein beta1 subunit (GABP-β1), mothers against decapentaplegic homolog 4 (SMAD4), WD Repeat Domain 77 (WDR77), and FK506-binding protein 51 (FKBP51) [18,19,20,21,22]. In vivo and in vitro loss of SIRT7 showed hyper-acetylation of p53 [23,24,25]. Moreover, knockdown of SIRT7 in both cells and mice enhanced the transcriptional activity of p53 on apoptosis and further activated the pro-apoptotic signaling pathway mediated by p53 [24,26,27]. However, other reports indicated that SIRT7 lacks p53 deacetylation activity [14].

The tumor suppressor p53 plays a critical role in cellular processes, including cell growth, cell differentiation, and cell death, in response to a wide range of cellular stresses [28]. In this study, we found that acetylation levels of STRAP may be involved in the regulation of p53-mediated signaling pathways. We demonstrated that SIRT7 deacetylates STRAP directly and influences the interaction between STRAP and p53. Importantly, mutation of the acetylation sites of STRAP leads to changes in p53 activity and stability.

## 2. Results

### 2.1. SIRT7 Interacts with STRAP

**Figure 1:**
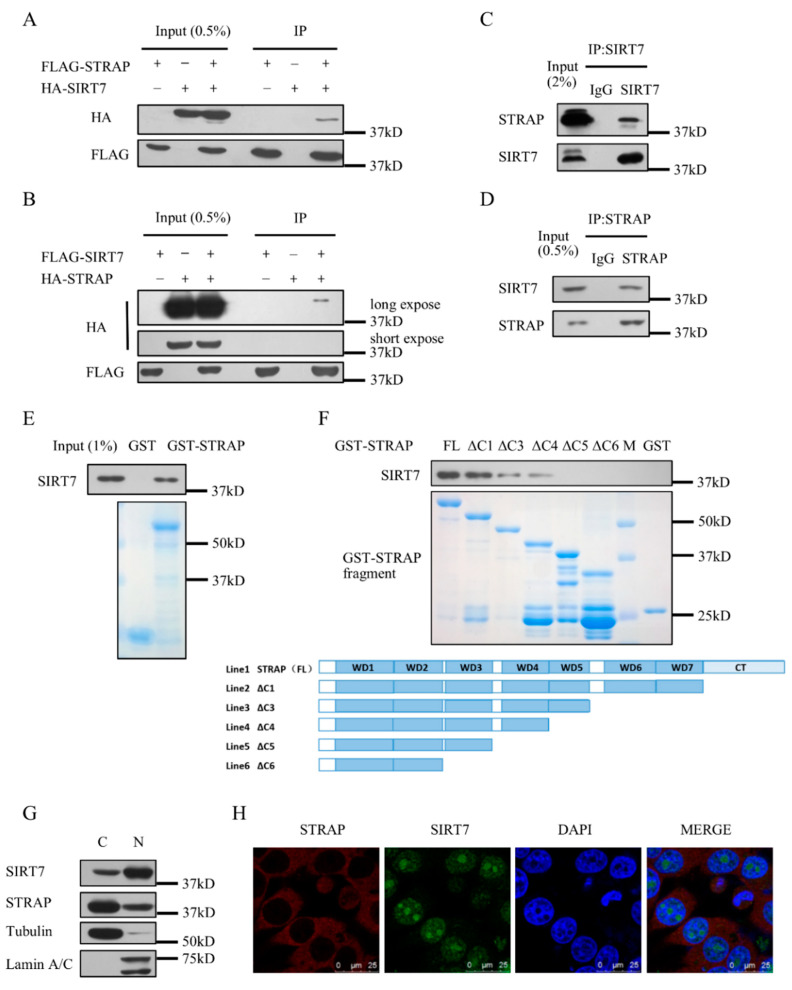
Serine-threonine kinase receptor-associated protein (STRAP) interacts with sirtuin7 (SIRT7) in vivo and in vitro. (**A**,**B**) STRAP interacts with SIRT7 in vivo. HEK293T cells were co-transfected with tagged STRAP and tagged SIRT7. Whole-cell lysates were immune-precipitated with M2 beads and analyzed by Western blotting with indicated antibodies. (**C**,**D**) Co-immunoprecipitation of STRAP with SIRT7. Whole HEK293T cell lysates were immune-precipitated with control IgG, anti-SIRT7 or anti-STRAP antibodies, and analyzed by Western blotting with anti-STRAP or anti-SIRT7, respectively. (**E**,**F**) STRAP interacts with SIRT7 in vitro. GST fusion proteins were generated for full-length STRAP (FL) and different length of truncated STRAP, ΔC1, ΔC3, ΔC4, ΔC5, ΔC6. SIRT7 protein was purified from 293T cells. GST-pull-down assays were performed as described in Material and Methods. (**G**) Subcellular localization of STRAP and SIRT7, revealed by biochemical fractionation. HCT116 cell lysates were analyzed by antibodies as indicated. (**H**) Co-localization of STRAP and SIRT7. HCT116 cells were immune-stained using anti-STRAP or anti-SIRT7 antibodies, followed by Alexa Fluor-594 anti-mouse antibody (for STRAP, in red) or Alexa Fluor-488 anti-rabbit antibody (for SIRT7, in green) and then examined using confocal microscopy. The yellow color in the merged image represents the co-localization of STRAP and SIRT7. Representative images are shown.

In our previous report, we purified the SIRT7 complex by immunoprecipitation and mass spectrometry. STRAP was found in the complex [29]. To verify the interaction between STRAP and SIRT7, we overexpressed tagged STRAP and tagged SIRT7 into HEK293T cells and performed co-immunoprecipitation (Co-IP) assays. The results showed that SIRT7 can interact with STRAP (Figure 1A), and vice versa (Figure 1B). STRAP–SIRT7 interaction was further supported by the Co-IP of endogenous STRAP and SIRT7 (Figure 1C,D). To confirm this interaction, we purified Glutathione S-transferase (GST) fusion proteins of full-length STRAP (WT) and different GST-tagged truncation of STRAP as indicated (Figure 1F). STRAP was divided into five segments according to its domains: ΔC1 (NT-WD7), ΔC3 (NT-WD5), ΔC4 (NT-WD4), ΔC5 (NT-WD3), ΔC6 (NT-WD2). SIRT7 can clearly be pulled down by GST-STRAP full length (Figure 1E). SIRT7 can also be pulled down by GST-STRAP (ΔC1), not GST-STRAP (ΔC3) or GST-STRAP (ΔC4) as strong as GST-STRAP (FL), indicating that SIRT7 mainly interacts independently and directly with STRAP at the WD region (Figure 1F). According to the reports, STRAP is mainly present in the cytoplasm, and SIRT7 is mainly present in the nucleus. We confirmed the subcellular localization of SIRT7 and STRAP in HCT116 cells by biochemical fractionation assay. STRAP was mainly present in the cytoplasm (C) but was also present in the nuclear fractions. Furthermore, SIRT7 was detected not only in the nucleus (N) but also in the cytoplasmic fractions (Figure 1G). We further performed immunofluorescence assay to detect subcellular localization of STRAP and SIRT7. Co-localization of STRAP and SIRT7 was observed in both cytoplasm and nucleus (Figure 1H). These results provide evidence that SIRT7 interacts with STRAP both in vivo and in vitro.

### 2.2. STRAP Is Acetylated at Lysines 147, 148, and 156

**Figure 2:**
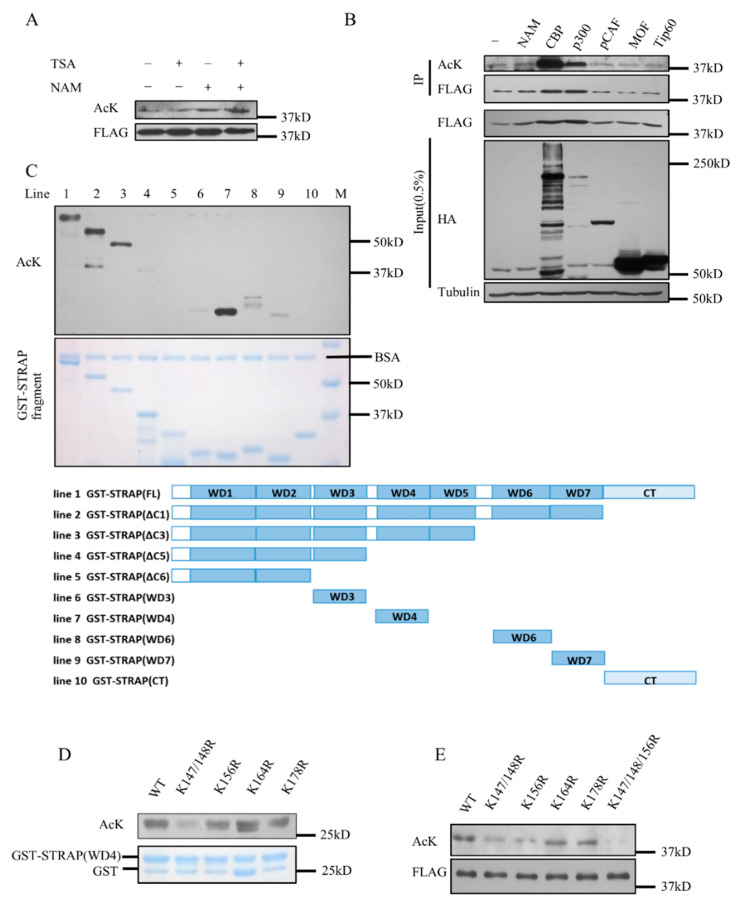
STRAP is acetylated at lysines 147, 148, and 156. (**A**) HEK293T cells were transfected with Flag-STRAP for 24 h and incubated with or without 1 μM TSA and/or 5 mM nicotinamide (NAM), as indicated, for an additional 6 h. An in vivo acetylation assay and Western-blotting analysis were performed. (**B**) HEK293T cells were co-transfected with plasmids containing Flag-STRAP and different HA (Hemagglutinin)-tagged acetyltransferases, CBP, p300, PCAF, hMOF, or Tip60. Whole-cell lysates were immune-precipitated with M2 beads and analyzed by Western blotting with indicated antibodies. (**C**) Ten types of GST-STRAP fusion proteins were used for in vitro acetylation assays. Western-blotting data (upper panel). Gel code blue staining (bottom panel). (**D**) GST-STRAP (WD4) Wild-type or the K to R mutant GST-STRAP (WD4) fusion proteins were used for in vitro acetylation assays. (**E**) HEK293T cells were transfected with wild-type or the indicated K to R mutant Flag-tagged STRAP constructs for 24 h and incubated with 1 μM TSA and 5 mM NAM for an additional 6 h. The levels of acetylation and total STRAP protein were detected after anti-Flag M2 immune-precipitation.

The STRAP–SIRT7 interaction indicated that STRAP could be a novel substrate of SIRT7. Therefore, we first examined whether STRAP could be acetylated. We used HDAC inhibitor Trichostatin A (TSA) and Sirtuins inhibitor nicotinamide (NAM) to detect the acetylation status of STRAP. STRAP acetylation was more significant in cells treated with NAM than in cells treated with TSA (Figure 2A), indicating that the major deacetylase of STRAP could be sirtuins rather than HDACs I, II, IV. We co-transfected Flag-STRAP with certain acetyltransferases, such as CBP, p300, pCAF, MOF, Tip60 into HEK293T cells and performed immune-precipitate assay. STRAP acetylation was detected when co-transfected with CBP or p300 in vivo (Figure 2B). Then we performed an in vitro acetylation assay using the GST-STRAP described in Figure 1F and some additional constructed fragments. The results showed that STRAP was mainly acetylated at the WD4 domain in vitro (Figure 2C). The WD4 domain contains five lysines at 147, 148, 156, 164, and 178 as possible targets for acetylation; hence we purified the GST-STRAP (WD4) WT or mutant K147/148R, K156R, K164R, K178R fusion proteins and performed the in vitro acetylation assay. Lysine residues 147/148, 156, 178 were detected by anti-pan-acetyl lysine (Figure 2D). We further mutated these lysines to arginines (K147/148R, K156R, K164R, K178R and 3KR), and as expected, the 3KR mutant acetylation level decreased significantly (Figure 2E). All these data indicated that STRAP is acetylated by CBP both in vivo and in vitro and that K147, K148, and K156 are the major acetylation sites of STRAP.

### 2.3. STRAP Is Deacetylated by SIRT7

**Figure 3:**
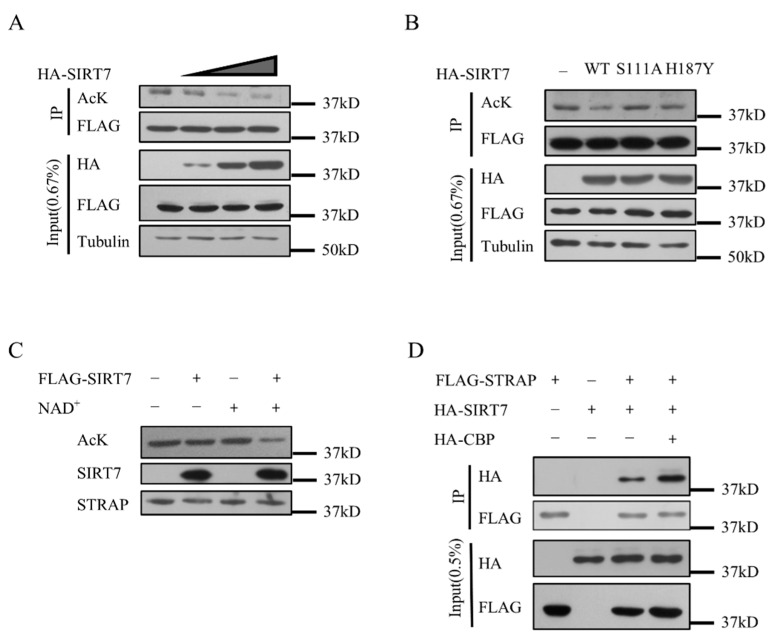
Sirt7 deacetylates STRAP in vivo and in vitro. (**A**) In vivo deacetylation assay for STRAP. HEK293T cells were co-transfected with Flag-STRAP, HA-CBP, or with increasing amounts of HA-SIRT7 plasmid, followed by deacetylation assays. (**B**) HEK293T cells were co-transfected with Flag-STRAP, HA-CBP and empty vector or with HA-SIRT7 (WT), HA-SIRT7-S111A (S111A) or HA-SIRT7-H187Y (H187Y), followed by deacetylation assays. (**C**) In vitro deacetylation assay for STRAP. High acetylated Flag-STRAP and HA-SIRT7 were purified from HEK293T cells, followed by in vitro deacetylation assays, in the presence of NAD+ or not. (**D**) CBP can enhance the interaction of SIRT7 and STRAP. Western blotting of whole-cell extracts and co-immunoprecipitates with the anti-Flag M2 beads from cells transfected with or without Flag-STRAP/HA-SIRT7/HA-CBP as indicated.

Based on the previous results, we then explored whether SIRT7 deacetylates STRAP. We first co-transfected Flag-STRAP and different amounts of HA-SIRT7 into HEK293T cells. Western blotting showed that STRAP acetylation levels decreased with increasing amounts of SIRT7 transfection (Figure 3A). We then performed the in vitro deacetylation assay to confirm whether SIRT7 directly deacetylates STRAP. Acetylated STRAP was purified and incubated under different conditions.

Interestingly, the acetylation level of STRAP decreased with SIRT7 and NAD+ co-incubation (Figure 3C), suggesting SIRT7 is a direct deacetylase for STRAP. On the other hand, we co-transfected STRAP with SIRT7-WT, SIRT7-S111A, or SIRT7-H187Y and detected STRAP acetylation levels. The catalytically inactive SIRT7 mutant (S111A/H187Y) could not deacetylate STRAP (Figure 3B). Moreover, the interaction of STRAP and SIRT7 was enhanced by the co-expression of histone acetyltransferase CBP (Figure 3D). Thus, STRAP is a novel substrate for SIRT7, which can deacetylate STRAP both in vivo and in vitro, dependent on SIRT7 catalytic activity.

### 2.4. Acetylation of STRAP Regulates p53-Mediated Transcription

**Figure 4:**
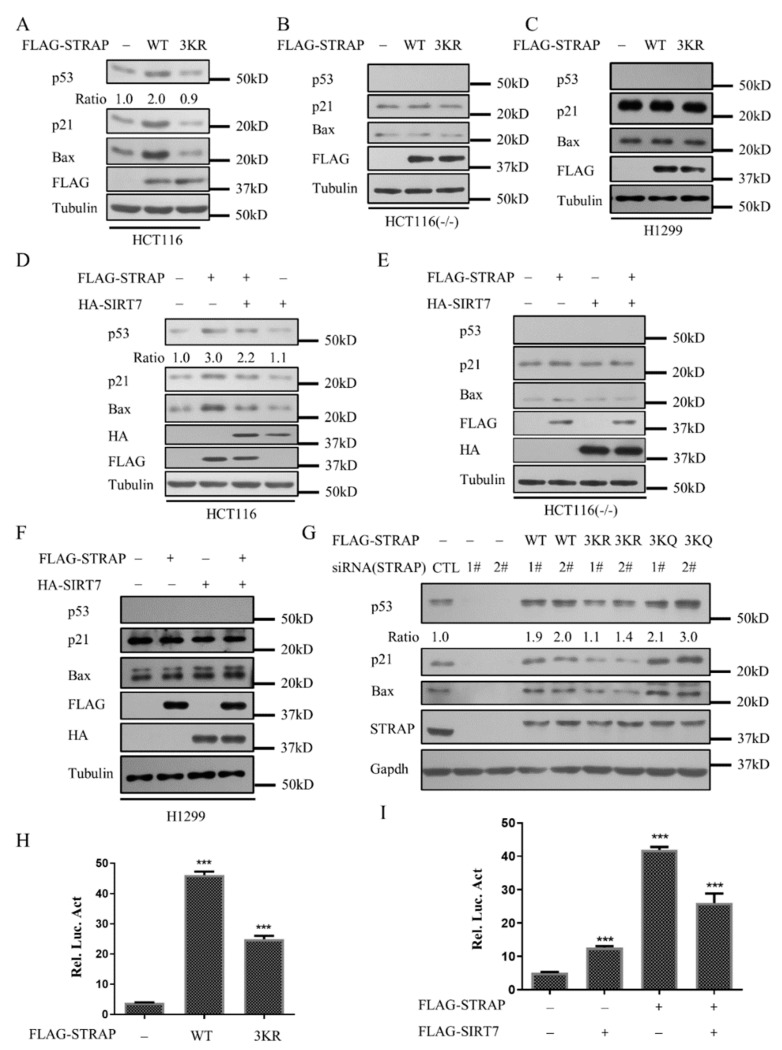
Acetylation of STRAP regulates p53-mediated transcription. (**A**–**C**) Effect of STRAP acetylation on the expression of p53 target genes. HCT116 (wild-type), H1299, and HCT116 (p53 -/-) cells were transfected with the indicated plasmid vectors expressing a control vector (Vector), STRAP-WT (WT), or STRAP-3KR (3KR). Whole-cell lysates were analyzed by Western blotting with indicated antibodies. (**D**–**F**) HCT116 (wild-type), H1299, and HCT116 (p53 -/-) cells were co-transfected with or without the plasmid FLAG-STRAP or HA-SIRT7. Whole-cell lysates were analyzed by Western blotting. (**G**) Modulation of p53 target genes by knockdown of STRAP. HCT116 cells were transiently transfected with 100 nM control siRNA (CTL) or STRAP-specific siRNA (1# and 2#) as indicated for 24 h and then transiently transfected with vector (Vector), STRAP-WT (WT), STRAP-3KR (3KR), or STRAP-3KQ (3KQ) for 24 h. Whole-cell lysates were also subjected to Western-blotting analysis using indicated antibodies. (**H**) Regulation of p53-mediated transcription by STRAP acetylation. HCT116 cells were transfected with STRAP-WT (WT) or STRAP-3KR (3KR), as indicated, together with 40 ng pRL-TK internal control. (**I**) HCT116 cells were co-transfected with or without STRAP (WT) or SIRT7, as indicated, together with 40 ng pRL-TK internal control. Fold activation relative to the control un-transfected samples was calculated, and the standard deviations are less than 5%. The data are representative of at least three independent experiments. *** *p* < 0.001.

To explore the role of STRAP acetylation in the regulation of p53-mediated signaling pathway, HCT116 (wild-type), HCT116 (p53 -/-), and H1299 cells were transfected with STRAP-WT (WT) and STRAP-3KR (3KR), respectively. In HCT116 cells, expression of STRAP-WT caused the obvious upregulation of p53 as well as its targets, including p21 and Bax. Conversely, STRAP-3KR decreased this effect (Figure 4A). However, this effect was abolished in HCT116 (p53 -/-) and H1299 cells (Figure 4B,C). The above results indicated that the STRAP acetylation plays a key role in stimulating p53 activity. Similar results were also obtained with HCT116 (wild-type), HCT116 (p53 -/-), and H1299 cells that transfected with or without STRAP-WT (WT)/SIRT7 (Figure 4D–F). These results indicated that SIRT7 mediated STRAP deacetylation decreases p53 activity. Consistently, p53, p21, and Bax expression were reduced with the transfection of STRAP-specific siRNA. However, the levels of p53, p21, Bax expressions were back various degrees by transfection with STRAP-WT, STRAP-3KR, or STRAP-3KQ (Figure 4G). We also performed luciferase assay to confirm that STRAP acetylation levels regulate the p53-mediated transcription. Similar to the Western-blotting results, STRAP-3KR, as well as STRAP deacetylated by SIRT7, reduced the upregulation of p53 relative to STRAP-WT (Figure 4H,I). Together, these data indicated that SIRT7 mediated STRAP acetylation levels influence p53 activity.

### 2.5. Acetylation of STRAP Modulates p53 Stability

**Figure 5:**
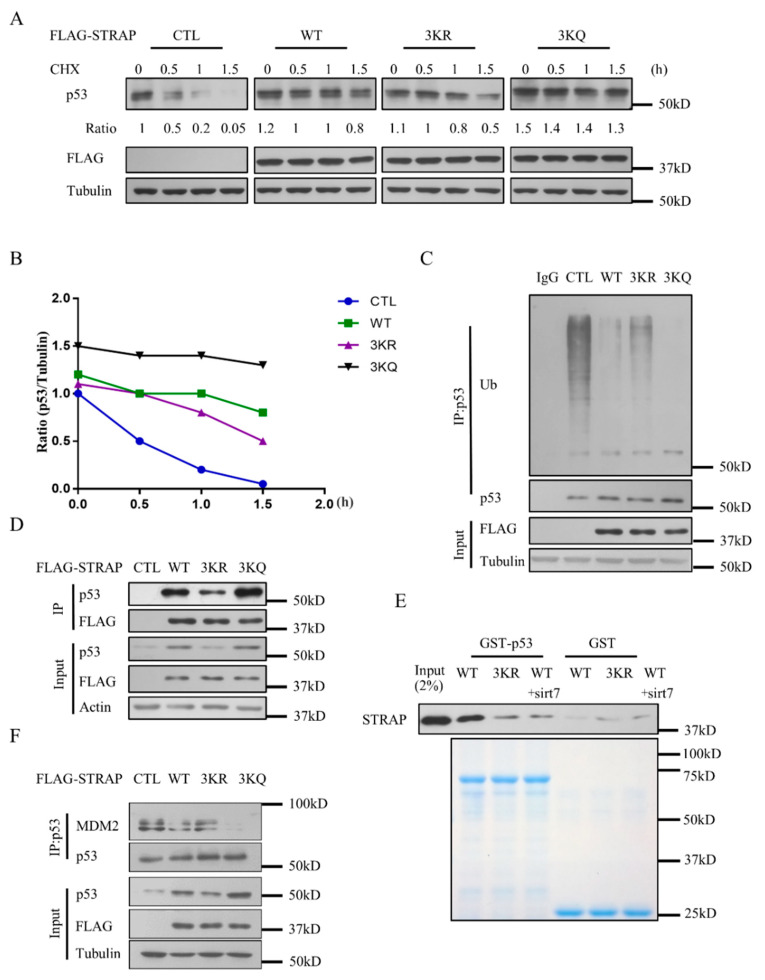
Modulation of p53 stability by STRAP acetylation. (**A**) Measurement of p53 stability by Western blotting with an anti-p53 antibody. HCT116 cells were transiently transfected with pcDNA3.1-Flag empty vector (Vector), STRAP-WT (WT), STRAP-3KR (3KR), or STRAP-3KQ (3KQ) for 24 h. Time intervals indicate the number of hours after cycloheximide (CHX) treatment (100 μg/mL). Whole-cell lysates were analyzed by Western blotting with indicated antibodies. (**B**) Line graph indicating the measured p53 levels under each condition determined by scanning the p53 bands. (**C**) Determination of p53 ubiquitination. HCT116 cells were transfected with pcDNA3.1-Flag empty vector (Vector), STRAP-WT (WT), STRAP-3KR (3KR), or STRAP-3KQ (3KQ), as indicated, together with HA-tagged ubiquitin (Ub). Whole-cell lysates were immune-precipitated with control IgG, anti-p53 antibody, and precipitated proteins were detected by an anti-HA antibody to determine the level of p53 ubiquitination. (**D**) STRAP interacts with p53 in vivo. HCT116 cells were transfected with pcDNA3.1-Flag empty vector (Vector), STRAP-WT (WT), STRAP-3KR (3KR), or STRAP-3KQ (3KQ) for 24 h. Whole-cell lysates were immune-precipitated with M2 beads and analyzed by Western blotting with indicated antibodies. (**E**) STRAP interacts with p53 in vitro. Flag-STRAP (WT), Flag-STRAP (3KR), and Flag-STRAP (WT) with SIRT7 were purified from HEK293T cells. GST fusion proteins were generated for p53. GST-pull-down assays were carried out as described in Material and Methods. (**F**) Amount of p53-bound Mdm2. HCT116 cells were transfected with pcDNA3.1-Flag empty vector (Vector), STRAP-WT (WT), STRAP-3KR (3KR), or STRAP-3KQ (3KQ) for 24 h, analyzed by Western blotting with indicated antibodies.

We further investigated the regulation mechanism of STRAP acetylation on p53. Half-life assay was performed by using empty vector (Vector), STRAP-WT (WT), STRAP-3KR (3KR), and STRAP-3KQ (3KQ) constructs. As compared with the control, the expression of STRAP-WT, STRAP-3KR, or STRAP-3kQ all partly increased the p53 half-life in HCT116 cells, while STRAP-3KR decreased the p53 half-life as compared with STRAP-WT. The result showed that deacetylated STRAP can reduce the stability of p53 relative to wild-type STRAP (Figure 5A,B). We then studied the role of STRAP acetylation in p53 ubiquitination. Expression of STRAP-WT, STRAP-3KR, or STRAP-3kQ all significantly decreased p53 ubiquitination levels, whereas the p53 ubiquitination levels were increased with the expression of STRAP-3KR as compared with STRAP-WT (Figure 5C). The interaction between p53 with deacetylated STRAP was further confirmed by the Co-IP and GST pull-down assay, with 3KR showing significantly reduced interaction with p53 (Figure 5D,E). We further confirmed the amount of p53-bound Mdm2 by Co-IP assay, transfected with WT or 3KQ showed significantly reduced interaction with p53 (Figure 5F). Together, these data indicated that STRAP acetylation affects its interaction with p53, reducing p53 ubiquitination levels and increasing its half-life.

### 2.6. STRAP Acetylation Levels Are Regulated by 5-FU

**Figure 6:**
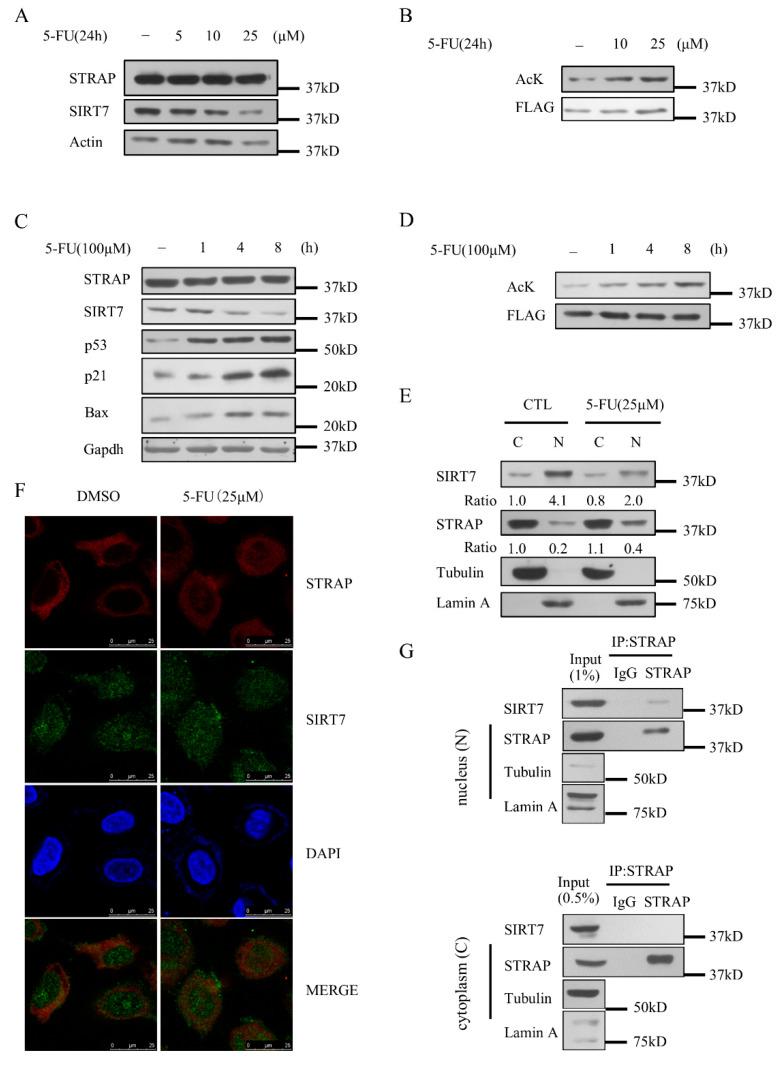
STRAP acetylation levels are specifically modulated by 5-fluorouracil (5-FU). (**A**) Western blotting for STRAP or SIRT7 endogenous levels in HCT116 cells following 5-FU treatment at 5, 10, 25 μM for 24 h. (**B**) Increases in STRAP acetylation status upon 5-FU treatment. Flag-STRAP was immune-precipitated from HCT116 cells treated with DMSO, 10 μM, 25 μM 5-FU for 24 h, and eluted for Western blotting with an anti-pan-acetylation antibody. (**C**) Western blotting for STRAP or SIRT7 endogenous levels in HCT116 cells following 100 μM 5-FU treatment for 0, 1, 4, and 8 h. (**D**) Increases in STRAP acetylation status upon 5-FU treatment. Flag-STRAP was immune-precipitated from HCT116 cells treated with 100 μM 5-FU treatment for 0, 1, 4, and 8 h and eluted for Western blotting with an anti-pan-acetylation antibody. (**E**) Subcellular localization of STRAP and SIRT7 treated with or without 5-FU revealed by biochemical fractionation. U2OS cell lysates were analyzed by antibodies as indicated. (**F**) Co-localization of STRAP and SIRT7. U2OS cells were immune-stained using anti-STRAP or anti-SIRT7 antibodies, followed by Alexa Fluor-594 anti-mouse antibody (for STRAP, in red) or Alexa Fluor-488 anti-rabbit antibody (for SIRT7, in green) and then examined using confocal microscopy. Yellow staining in the merged images shows co-localization between STRAP and SIRT7 after 5-FU treatment. Representative images are shown. (**G**) Co-immunoprecipitation (Co-IP) of STRAP with SIRT7 in the nucleus (N) and cytoplasm (C) upon 5-FU treatment. Treated HCT116 cells with 25 μM 5-FU for 24 h, separated cytoplasm and nucleus by biochemical fractionation, then immune-precipitated with and analyzed by antibodies as indicated.

A recent report showed that 5-fluorouracil (5-FU) induces radio-sensitivity via SIRT7 degradation, which promotes cell death during cancer cell radiotherapy [30]. To analyze the effect of 5-FU on STRAP, we first exposed HCT116 cells to 5-FU and analyzed the protein expression levels of SIRT7 and STRAP. SIRT7 levels decreased in a time and dose-dependent mode upon 5-FU treatment, whereas there was no marked change in STRAP following any of the treatment conditions (Figure 6A,C). We next explored whether STRAP acetylation was regulated by 5-FU. 5-FU treatment resulted in time- and dose-dependent induction of STRAP acetylation (Figure 6B,D). These results suggest that 5-FU increased the acetylation levels of STRAP and had no effect on the expression of STRAP. Combining these results, we confirmed the subcellular localization of SIRT7 and STRAP in U2OS cells upon 5-FU treatment by biochemical fractionation assay [31]. We observed that 5-FU treatment led to an increase in STRAP and a decrease in SIRT7 in the nuclear fraction (Figure 6E). The subcellular distribution of STRAP and SIRT7 upon 5-FU treatment was further validated by immunofluorescence assay. We observed the co-localization of STRAP and SIRT7 in both cytoplasm and nucleus (Figure 6F). We confirmed the STRAP–SIRT7 interaction in the nucleus (N) and cytoplasm (C) by biochemical fractionation assay upon 5-FU treatment (Figure 6G). Taken together, 5-FU treatments increased the acetylation levels of STRAP, without affecting its protein levels and influenced the subcellular distribution of STRAP.

## 3. Discussion

STRAP is known as a negative regulator in the TGF-β signaling pathway, participating in the regulation of cell growth, cell differentiation, and apoptosis in response to various stresses [4]. Here, we identified that STRAP is a novel target of SIRT7. STRAP is acetylated at K147, K148, and K156 by the acetyltransferases CBP/p300, and such acetylation is reversed by the deacetylase SIRT7. We demonstrated that 5-FU treatment led to the degradation of SIRT7, thereby disrupting the interaction of STRAP with SIRT7 and increasing STRAP acetylation. STRAP-3KR, which mimics hypoacetylated STRAP, specifically impaired the ability of the protein to interact with p53. Our data suggest that the acetylation status of STRAP plays an important role in p53 activity and stability.

STRAP acetylation has not been reported previously. A recent study revealed that STRAP stability and activity is mainly regulated by phosphorylation at threonine and serine residues [5]. Our study showed that STRAP was primarily acetylated by CBP/p300 at K147, K148, and K156, and deacetylated by SIRT7 (Figure 2 and Figure 3). In unstressed cells, STRAP is found mainly in the cytoplasm, with only a small proportion in the nucleus. Strikingly, upon 5-FU treatment, some cytoplasm fraction of STRAP was rapidly translocated into the nucleus. The 5-FU-induced degradation of SIRT7 and nuclear translocation of STRAP increased the extent of STRAP acetylation (Figure 6B,D,E). We analyzed the nuclear localization sequence of STRAP on the cNLS mapper website (http://nls-mapper.iab.keio.ac.jp/cgi-bin/NLS_Mapper_form.cgi) [32]. We obtained two sequences from the analysis, “GHTRPVVDLAFSGITPYGYFLISACKDGKP” located at STRAP-WD1 and “ELAKPKIGFPETTEEELEEIASENSDCIFPSAP” located at STRAP-CT. Additional experiments are required to clarify the mechanism of STRAP nuclear translocation. On the other hand, our results showed that SIRT7 levels decreased upon 5-FU treatment, but no translocation occurred (Figure 6E). However, in contrast to our results, one previous study found that some nucleus SIRT7 was translocated into the cytoplasm after the addition of doxorubicin or other DNA-damaging agents [31]. The reasons for such discrepancies are not clear, and further studies are required to resolve these discrepancies.

STRAP is considered a prognostic factor [33] due to the association between tumor stage and the protein levels. However, the role of STRAP in tumor progression remains controversial. On the one hand, Datta et al. showed that STRAP may provide growth advantages for tumor cells, supporting the oncogenic function of STRAP [1,8,9,34]. On the other hand, recent studies suggested that STRAP participates in cell death and proliferation through different signaling pathways [5,12,35]. However, the tumor-suppressive effects of STRAP have not yet been confirmed, although the pro-apoptotic function of STRAP was observed in cancer cells. Our results showed that STRAP could activate p53 alone (Figure 4), and STRAP acetylation affects its interaction with p53 and stability of p53 (Figure 5). Whether STRAP affects p53-mediated apoptosis needs further exploration.

During our investigation of the functions of STRAP acetylation, we also tried to examine its influence on the TGF-β pathway. However, we found that the STRAP acetylation neither affects its interaction with Smad7 nor causes any change in the TGF-β signaling pathway. It is interesting to see the differential regulation on STRAP acetylation within p53 and TGF-β signaling. It is possible that STRAP acetylation targets the p53 pathway and other post-translational modifications, such as phosphorylation, ubiquitination regulates TGF-β signaling pathway. Further investigation into the differential regulation of STRAP post-translational modifications on different targets and its regulating mechanisms would be worthwhile.

SIRT7 regulation of p53 activity has been reported by several groups with differing controversial results. In vivo and in vitro loss of SIRT7 showed hyper-acetylation of p53, increases the rate of apoptosis, thereby promoting cell survival [23,24,25]. The knockdown of SIRT7 enhanced the transcriptional activity of p53 toward apoptosis and activated the p53-mediated pro-apoptotic signaling pathway [24,26]. However, other reports indicated that SIRT7 lacked p53 deacetylation activity [14,27]. Recently, overexpression of SIRT7 led to increased p53 stability, but SIRT7 does not deacetylate p53 in vitro or in HT1080 or NHF cells [36]. Our findings on SIRT7 modulating p53 function through deacetylating STRAP added new insight into SIRT7-p53 regulation and reconciled the controversial studies.

## 4. Materials and Methods

### 4.1. Cell Culture

HEK293T, HCT116 (wild type), HCT116 (p53 -/-), H1299, and U_2_OS cells were cultured in DMEM (Invitrogen), supplemented with 10% (*v*/*v*) FBS, 100 U/mL penicillin/streptomycin, and incubated at 37 °C in humidified air with 5% CO2. The HCT116 cell line was obtained from Weiguo Zhu. The U_2_OS cell line was provided by Junjie Hu.

### 4.2. Antibodies, Plasmids, and Small Interfering RNA

The following primary antibodies were used: The mouse antiSIRT7 (sc-365344), rabbit anti-SIRT7 (sc135055), anti-p53 (FL) (sc-6243), anti-p53 (DO-1) (sc-126), anti-p21 (sc-271532), anti-actin (sc-8432) and anti-tubulin (sc-73242) were purchased from Santa Cruz Biotechnology (Dallas, TX, USA). The anti-STRAP (18277-1-AP) was purchased from Proteintech (Wuhan, China), the anti-GAPDH (#5174S) was purchased from Cell Signaling Technology (Danvers, MA, US), the anti-FLAG (F3165) was purchased from Sigma–Aldrich (St. Louis, MO, US), the anti-HA (#26183) was purchased from Pierce (Rockford, Illinois, US), the anti-pan-acetyllysine (#9441L) was purchased from Cell Signaling Technology (Danvers, MA, US).pcDNA3.1-Flag/HA-STRAP/SIRT7 was obtained by subcloning STRAP/SIRT7 sequence into pcDNA3.1-Flag/HA vector. The mutant constructs were generated by PCR amplification of STRAP/SIRT7 with specific primers. STRAP full-length (FL), STRAP (ΔC1, ΔC3, ΔC4, ΔC5, ΔC6, WD3, WD4, WD6, WD7, CT) were subcloned into the pGEX4T-3 vector.

STRAP siRNA 1#: gggugcaacacugaauaag; siRNA 2#: uuacgcauauaugacuuga (Genepharma, Suzhou, China).

### 4.3. Co-Immunoprecipitation

Protein/protein interaction was evaluated by Co-IP according to standard methods. Cell pellets were lysed in BC100 buffer (20 mM Tris-HCl (pH 7.9), 0.2% NP-40, 100 mM NaCl, and 20% glycerol) containing 1 mM Dithiothreitol (DTT), 1 mM Phenylmethanesulfonyl fluoride (PMSF), and protease inhibitor cocktail (Sigma–Aldrich, St. Louis, MO, US), centrifuged, and used for immune-precipitation. Western blotting was then performed to detect protein expression, as indicated in immune-precipitates.

### 4.4. GST Pull-Down Assay

For the pull-down assay, 1 μg of GST, GST-STRAP, or GST-p53 protein was incubated with Flag-tagged SIRT7/STRAP protein in 100 μL interaction buffer at 4 °C overnight in BC100, then incubated with glutathione–sepharose beads (50% slurry) for an additional 2 h. Bound GST fusion proteins were then resolved by SDS-PAGE and analyzed by Western blotting using antibodies as indicated.

### 4.5. Acetylation/Deacetylation Assay In Vivo

For the acetylation assay, cells were transfected with STRAP alone or co-transfected with STRAP and CBP for 24 h, treated with 1 μM TSA and 5 mM NAM (Sigma–Aldrich, St. Louis, MO, USA) for an additional 6 h before harvest. Cell extracts were incubated with anti-Flag M2 beads, and bound proteins were analyzed by Western blotting. For the deacetylation assay, cells were transfected with STRAP alone or co-transfected with STRAP and different amount of SIRT7 for 24 h, whole-cell lysates were prepared in BC100 supplemented with protease inhibitors. Cell extracts were incubated with anti-Flag M2 beads, and immune-precipitated proteins were analyzed with different antibodies by Western blotting.

### 4.6. In Vitro Acetylation Assay

The in vitro reactions were performed as described [29]. Recombinant 2 μg GST or GST fusion protein was incubated with 1 μg HA-CBP purified from 293T cells in a 30 μL system containing acetylation reaction buffer A [200 mM N-2-Hydroxyethylpiperazine-N-2-Ethane Sulfonic Acid (HEPES) (pH 8.0), 0.1 mM Ethylene Diamine Tetraacetic Acid (EDTA), 10 mM PMSF, 10 mM DTT, 170 nmol/L acetyl-CoAcarboxylase). Reactions were completely mixed and incubated at 37 °C for 2 h, the protein levels were represented by Coomassie blue staining, and the acetylation levels were analyzed by Western blotting.

### 4.7. In Vitro Deacetylation Assay

The in vitro reactions were performed as described [29]. Purified acetylated Flag-STRAP was incubated with or without purified HA-SIRT7 as indicated at 37 °C for 2 h in the deacetylation reaction buffer B [100 mM NaCl, 4 mM MgCl2, 50 uM NAD+ (Sigma–Aldrich, St. Louis, MO, USA), 10% glycerol, 1 mM DTT]. The reactions were resolved by SDS-PAGE and analyzed by Western blotting using antibodies specific for acetylated STRAP.

### 4.8. Luciferase Reporter Assay

A luciferase reporter assay was performed, as described [13]. HCT116 cells were transfected with the p53-luciferase reporter plasmid and pRL-TK plasmid, along with the appropriate plasmids, as indicated. The control plasmids were added to sustain equal amounts of total DNA. Luciferase activity was monitored with a luciferase assay kit (Promega, Madisoon, WI, USA) following the manufacturer’s instructions. The firefly luciferase activity was normalized with the value of the corresponding renilla luciferase activity, and the ratio (*n* = 3, mean ± S.D.) was statistically analyzed.

### 4.9. In Vivo Ubiquitination Assay

A ubiquitination assay was performed, as described [13]. HCT116 cells were transfected with Flag-STRAP or mutants, and HA-tagged ubiquitin plasmids indicated. After 24 h, 10 mg/mL MG132 was applied to the cells for 4 h before they were collected, and then lysed in 500 μL BC100 buffer. An immunoprecipitation of protein was performed with anti-p53 antibody. Bound protein was subjected to SDS-PAGE and Western blotting.

### 4.10. Immunofluorescence Staining

For all localization experiments, HCT116 cells were cultured on glass coverslips treated with or without 5-FU at 40–60% confluence. The cells were fixed in 4% paraformaldehyde for 10 min at Room temperature (RT), and then washed with PBS plus 0.2% Triton X-100 for 10 min and blocked for 1 h at 4 °C. Primary antibodies used were anti-STRAP (1:200) or anti-SIRT7 (1:200), and secondary antibodies used were Alexa Fluor-488 anti-mouse (1:1000) or Alexa Fluor-594 anti-rabbit (1:1000). Cell nuclei were stained with 4’,6-diamidino-2-phenylindole (DAPI) (Sigma, St. Louis, MO, US). Fluorescent images were taken and analyzed using a confocal laser microscope (Leica).

## 5. Conclusions

In summary, we have shown that STRAP can be acetylated by CBP and deacetylated by SIRT7. Moreover, following 5-FU treatment, STRAP is mobilized from the cytoplasm to the nucleus and promotes STRAP acetylation. Our finding on the regulation of STRAP links p53 with SIRT7 influences p53 activity and stability.
